# Annual acknowledgement of manuscript reviewers

**DOI:** 10.1186/s13012-016-0391-9

**Published:** 2016-03-11

**Authors:** Anne Sales, Michel Wensing

**Affiliations:** 1 Implementation Science, Department of Veterans Affairs and University of Michigan, Ann Arbor, USA; 2 Implementation Science, University of Heidelberg, Heidelberg, Germany

## Abstract

The editors of *Implementation Science* would like to thank all our reviewers who have contributed to the journal in Volume 10 (2015).

**Attila Altiner**

Germany

**Sarah Amador**

UK

**Stephanie Archer**

UK

**David Aron**

USA

**Lou Atkins**

UK

**Luciana Ballini**

Italy

**Melanie Barwick**

Canada

**Subal C Basak**

India

**Emma Beard**

UK

**Raj Behal**

USA

**Aminu K Bello**

Canada

**Jonathan Benn**

UK

**Fiona Beyer**

UK

**Jean Joel Bigna**

Cameroon

**Christian Blickem**

UK

**Annette Boaz**

UK

**Andrew Booth**

UK

**Alison Booth**

UK

**Anne-Marie Bostrom**

Sweden

**Paul Bowie**

UK

**Raymond Boyle**

USA

**Jeffrey Braithwaite**

Australia

**Jamie Brehaut**

Canada

**John Brodersen**

Denmark

**Carinne Brody**

USA

**Melissa Brouwers**

Canada

**Todd Brown**

USA

**C Hendricks Brown**

USA

**Ross Brownson**

USA

**Mathieu Bujold**

Canada

**Ben Byrne**

UK

**Guy Cafri**

UK

**Danielle Campbell**

Australia

**James Cane**

UK

**Diane Carpenter**

UK

**Enrique Castro-Sánchez**

UK

**David Chambers**

USA

**Sarah Chapman**

UK

**Esmita Charani**

UK

**Janita Pc Chau**

Hong Kong

**Navila Talib Chaudhry**

UK

**Timothy R. Church**

USA

**Fiona Clement**

Canada

**Deena Costa**

USA

**Janet Curran**

Canada

**Geoffrey Curran**

USA

**Kristina Curtis**

UK

**Laura Damschroder**

USA

**Kristin Danko**

Canada

**Frances Dark**

Australia

**James Dearing**

USA

**Brendan Delaney**

UK

**Maureen Dobbins**

Canada

**Rowena Dolor**

USA

**Sandra Dunn**

Canada

**Angela Durey**

Australia

**Carolyn Ehrlich**

Australia

**Jon Emery**

Australia

**Nick Emmel**

UK

**Lynne Emmerton**

Australia

**Eleonora Feletto**

Australia

**Deborah Fenlon**

UK

**Rashida Ferrand**

UK

**Tracy Finch**

UK

**Diane Finegood**

Canada

**Ellen Fineout-Overholt**

USA

**Jill Francis**

UK

**Alec Fraser**

UK

**Atle Fretheim**

Norway

**Tony Fryer**

Uruguay

**John Gabbay**

UK

**Anna Gagliardi**

Canada

**Liz Glidewell**

UK

**Rachael Gooberman-Hill**

UK

**Lucy Goulding**

UK

**Ian Graham**

Canada

**Eric Granholm**

USA

**Carla A. Green**

USA

**Karen Grimmer**

Australia

**Lisa Gugglberger**

Austria

**Stephen Halloran**

UK

**Ilana Halperin**

Canada

**Alison Hamilton**

USA

**Margaret Handley**

USA

**Steven Hanna**

Canada

**Wendy Hardeman**

UK

**Matire Harwood**

New Zealand

**Henna Hasson**

Sweden

**Susanne Hempel**

USA

**Uy Hoang**

UK

**Justus Hofmeyr**

South Africa

**Bev J Holmes**

Canada

**Caroline Homer**

Australia

**Sarah Horwitz**

USA

**Susan Huckson**

Australia

**Claire Hulme**

UK

**Dominic Hurst**

UK

**Bettina Husebo**

Norway

**Alison Hutchinson**

Australia

**Emily Hyle**

USA

**Denise Hynes**

USA

**Sylvia Hysong**

USA

**Alfonso Iorio**

Canada

**Theodore Iwashyna**

USA

**Susan Jack**

Canada

**Claire Jackson**

Australia

**George Jackson**

USA

**Justin Jagosh**

Canada

**Roberta James**

UK

**Caroline Jones**

UK

**David Katz**

USA

**Michael Kelly**

UK

**Anne Kennedy**

UK

**Alec Knight**

UK

**Danica Knight**

USA

**Anita Kothari**

Canada

**Peter Lachman**

UK

**Rebecca Lawton**

UK

**Cara Lewis**

USA

**Clare Liddy**

Canada

**Chuan-Fen Liu**

USA

**Fabiana Lorencatto**

UK

**Laura Louison**

USA

**Lisa Lubomski**

USA

**Berit Lundman**

Sweden

**Aaron Lyon**

USA

**Michael Maciosek**

USA

**Kate Mandeville**

UK

**Carl May**

UK

**Tanya Mccance**

UK

**Ruth Mcdonald**

UK

**Brian Mckenna**

Australia

**James Mckenna**

UK

**Sandy Middleton**

Australia

**Norweeta Milburn**

USA

**Anne Miles**

UK

**Robin Miller**

UK

**Brian Mittman**

USA

**Kaelan Moat**

Canada

**Andrew Morden**

UK

**Leanne Morrison**

UK

**Elizabeth Murray**

UK

**Eleanor Murray**

UK

**Juliet Nabyonga-Orem**

Uganda

**Cate Nagle**

Australia

**Felix Naughton**

UK

**Per Nilsen**

Sweden

**Wynne Norton**

USA

**Ronan O'Carroll**

UK

**Maria O'Connell**

USA

**Hannah O'Rourke**

Canada

**Andy Oxman**

Norway

**Samuel Pannick**

UK

**Sara Paparini**

UK

**Sanjay Patel**

UK

**Mark Pearson**

UK

**Ricardo Perez-Cuevas**

Mexico

**Tahna Pettman**

Australia

**David Pilgrim**

UK

**Stephanie Polus**

Germany

**Henry Potts**

UK

**John Powell**

UK

**Byron Powell**

USA

**Justin Presseau**

Canada

**Enola Proctor**

USA

**Craig Ramsay**

UK

**Nicole Rankin**

Australia

**Tim Rapley**

UK

**Salman Rawaf**

UK

**Tom Reader**

UK

**Nicola Robinson**

UK

**Stefania Rodella**

Italy

**Scott Roesch**

USA

**Louise Rohrbach**

USA

**Milagros Rosal**

USA

**Meagen Rosenthal**

USA

**Laurence Roy**

Canada

**Jo Rycroft-Malone**

UK

**Euan Sadler**

UK

**Caroline Sanders**

UK

**Shannon Scott**

Canada

**Kate Seers**

UK

**Peter Selby**

Canada

**Nick Sevdalis**

UK

**Lion Shahab**

UK

**Kenneth Sherr**

USA

**Rahul Shidhaye**

USA

**Steve Sparks**

UK

**Janet Squires**

Canada

**Marla Steinberg**

Canada

**Aslak Steinsbekk**

Norway

**Rachel Tabak**

USA

**Martina Teichert**

Netherlands

**Aleksandra Torbica**

Italy

**Samson Tse**

Hong Kong

**Phil Ullrich**

USA

**Nigel Unwin**

Barbados

**Robin Urquhart**

Canada

**Thomas Valente**

USA

**Trudy Van Der Weijden**

Netherlands

**Ivaylo Vassilev**

UK

**Sten Vermund**

USA

**Ruth Vine**

Australia

**Joan Vlayen**

Belgium

**Horst Christian Vollmar**

Germany

**Adrian Wagg**

Canada

**Dawn-Marie Walker**

UK

**Lars Wallin**

Sweden

**Thomas Waltz**

USA

**Dianne Ward**

USA

**Rosie Webster**

UK

**Bryan Weiner**

USA

**Elizabeth West**

UK

**Nathaniel Williams**

USA

**Cameron Willis**

Canada

**Paul M Wilson**

UK

**Shannon Wiltsey Stirman**

USA

**Charles Wolfe**

UK

**Patricia Wright**

USA

**Fred Wulczyn**

USA

**Sally Wyke**

UK

**Jane Young**

Australia

